# Failure of pyriproxyfen at recommended application frequency and doses to control *Aedes* mosquitoes in Thailand

**DOI:** 10.1371/journal.pntd.0013042

**Published:** 2025-12-22

**Authors:** Thipruethai Phanichat, Vincent Corbel, Benedicte Fustec, Chamsai Pientong, Kesorn Thaewnongiew, Neal Alexander, Hans J. Overgaard

**Affiliations:** 1 Department of Medical Entomology, Faculty of Tropical Medicine, Mahidol University, Bangkok, Thailand; 2 MIVEGEC, Univ. Montpellier, IRD, CNRS, Montpellier, France; 3 Laboratório de Biologia, Controle e Vigilância de Vetores (LBCVIV), Instituto Oswaldo 9 Cruz (IOC)/ FIOCRUZ, Rio de Janeiro, Brazil; 4 Department of Biological Sciences, University of Notre Dame, Notre Dame, Indiana, United States of America; 5 Department of Microbiology, Khon Kaen University, Khon Kaen, Thailand; 6 HPV & EBV and Carcinogenesis Research Group, Khon Kaen University, Khon Kaen, Thailand; 7 Department of Disease Control, Office of Disease Prevention and Control, Region 7 Khon Kaen, Ministry of Public Health, Khon Kaen, Thailand; 8 MRC Tropical Epidemiology Group, London School of Hygiene and Tropical Medicine, London, United Kingdom; 9 Tropical Disease Research Center, Khon Kaen University, Khon Kaen, Thailand; 10 Faculty of Science and Technology, Norwegian University of Life Sciences, Ås, Norway; University of California Davis School of Veterinary Medicine, UNITED STATES OF AMERICA

## Abstract

**Background/objectives:**

Mosquito-borne diseases like malaria, dengue fever, and Zika virus remain global health concerns. Pyriproxyfen is effective in controlling mosquitoes by disrupting their development. This study seeks to assess pyriproxyfen’s ability to prevent *Aedes aegypti* emergence from water sources. It is part of a trial evaluating pyriproxyfen’s impact on reducing mosquito infestation and dengue transmission, verifying its persistence and effectiveness in real-world and laboratory conditions.

**Methods:**

The study was conducted in Khon Kaen province (northeastern region) and Prachuap Khiri Khan province (western region) of Thailand. We assessed pyriproxyfen residual effectiveness, inhibition of mosquito larval emergence and active ingredients among batches in a pyriproxyfen-based mosquito control trial in Khon Kaen. In Prachuap Khiri Khan we evaluated pyriproxyfen effectiveness across various water sources. The active ingredients in two pyriproxyfen batches were analyzed in a Sumitomo laboratory and in an independent laboratory.

**Results:**

Thirty days after field water containers were treated with pyriproxyfen the inhibition of mosquito larval emergence declined to ~60% and 60 days post-treatment the inhibition of emergence was just ~10%. Two batches of pyriproxyfen tested in the laboratory had > 85% inhibition of emergence and the active ingredient concentrations varied from 0.45-0.52%, close to the manufacturer’s specifications of 0.5%. In laboratory experiments, the inhibition of mosquito emergence of pyriproxyfen in different water sources started declining after 42 days. Rain- and groundwater had higher inhibition rates (20–30%) than tap water (~10%) after 98 days. Emergence inhibition rates correlated negatively with water pH (F(1,118) = 5.626, p < 0.001) and positively with total dissolved solids, conductivity, and salinity of the water (F(1,118) = 48.302, p < 0.001), (F(1,118) = 37.022, p < 0.001), and (F(1,118) = 36.699, p < 0.001), respectively.

**Conclusions:**

Pyriproxyfen failed to control *Aedes* mosquitoes at the recommended application frequency and doses in the field. The potential reasons for lack of effectiveness may be caused by environmental factors, such as pH, water source, and other water characteristics or social factors, such as homeowners’ behaviors and water storage practices. The study underscores the importance of understanding environmental and social factors to tailor application strategies and ensuring sustained efficacy through regular monitoring, particularly in diverse contexts.

## Author summary

We examined the effectiveness of pyriproxyfen, a chemical used to control immature mosquitoes by preventing their development, particularly focusing on the *Aedes aegypti* mosquito, which spreads diseases like dengue. The research was conducted in Thailand and aimed to evaluate how well pyriproxyfen prevents adult mosquitoes to emerge in different water sources and under real-world conditions. Field tests showed that pyriproxyfen’s ability to stop mosquito larvae from emerging decreased over time. After 30 days, the inhibition rate was about 60%, and by 60 days, it dropped to just 10%. In contrast, laboratory tests showed higher effectiveness, with over 85% inhibition. The effectiveness of pyriproxyfen also declined in various water sources, with rain and groundwater maintaining better inhibition rates than tap water. Environmental factors like water pH and dissolved solids also affected the effectiveness of pyriproxyfen. Additionally, human behaviors, such as water storage practices, may have influenced the results. Overall, the findings suggest that pyriproxyfen may not be effective enough in field conditions without adjustments to dosage and application strategies. Regular monitoring and consideration of both environmental and social factors are recommended to improve its performance in mosquito control efforts.

## Introduction

Several *Aedes* mosquito species, particularly those in the subgenus *Stegomyia* are responsible for transmitting viruses to humans. Globally, *Aedes aegypti* (L.) is a primary vector of dengue, chikungunya, Zika and yellow fever viruses. *Aedes albopictus* (Skuse) is regarded a secondary vector and is also important for transmitting these arboviruses. Both species are common in Thailand. They generally utilize household and peridomestic water storage containers for oviposition and development of immature stages. One key strategy for vector control is larval source management either by source reduction (habitat elimination) or container treatment with a chemical or biological agent. In Thailand, larval vector control generally consists of applying an inexpensive organophosphate insecticide (temephos) in water storage containers [1,2].

Effective larval control measures are critical for reducing vector populations and disease transmission. Traditional control strategies, such as the use of larvicides, face challenges due to the increasing prevalence of insecticide resistance among *Aedes* larvae. Studies in Thailand have reported widespread resistance to commonly used chemical insecticides, including temephos and pyrethroids, emphasizing the need for alternative vector control tools [3,4].

*Aedes aegypti* has developed operationally significant resistance to temephos and other compounds in Thailand [5] and other areas of Southeast Asia [6–8]. Alternative insecticides and compounds are needed and require validation as replacement chemicals. In the quest for effective mosquito control, pyriproxyfen has emerged as a promising tool. Pyriproxyfen is an insect growth regulator that disrupts the development of mosquitoes, preventing them from reaching maturity and reproducing. This compound exhibits a unique mode of action by mimicking juvenile hormone, interfering with the normal metamorphosis process in mosquitoes without affecting other non-target organisms [9]. The long-lasting effects of pyriproxyfen make it a valuable component in integrated mosquito management programs. Furthermore, its low toxicity to humans and other non-target species adds to its appeal as a safe and sustainable solution for mosquito control [10] and is recommended by WHO for vector control [11,12]. Pyriproxyfen has also been approved by WHO for use in drinking water storage containers [13]. Pyriproxyfen has been shown to be effective in controlling mosquito populations. A systematic review showed that pyriproxyfen granules effectively inhibited emergence by 90–100% for up to 90 days [14]. Pyriproxyfen is applied in various formulations, including sprays, pellets, and insecticide-treated nets. These formulations enable flexible and targeted application methods, allowing for customized mosquito control strategies based on local conditions. The versatility of pyriproxyfen makes it an integral part of integrated vector management approaches, complementing other control measures such as insecticide-treated bed nets and larvicides. *Aedes* mosquitoes in Thailand have shown various levels of susceptibility to pyriproxyfen. While the insect growth regulator has been effective in some cases [15,16], there have been reports of reduced efficacy [17], highlighting the importance of ongoing surveillance and the need for integrated vector management strategies. However, there is limited research evaluating its residual efficacy and effectiveness under real-world conditions in tropical regions like Thailand. Furthermore, most studies focus on laboratory-based evaluations or short-term trials, leaving gaps in understanding its long-term performance and effectiveness across different socio-environmental settings. This study aims to address these gaps by assessing the characterization and residual effectiveness of pyriproxyfen under field conditions in Thailand. By evaluating its performance in diverse environments, this research provides valuable insights into the factors influencing pyriproxyfen efficacy, which can guide its optimized application in larval source management.

In our cluster randomized controlled trial [18] the pyriproxyfen failed to perform as expected; [19] requiring a need to better understand the intrinsic and extrinsic causes of this failure. We intended to validate the effectiveness and persistence of pyriproxyfen in the field and laboratory. Our specific objectives were to: 1) determine the residual efficacy of pyriproxyfen in inhibiting the emergence of *Ae. aegypti* in water from key domestic artificial containers in the field; 2) determine the field efficacy of two batches of pyriproxyfen in inhibiting *Ae. aegypti* emergence; 3) determine the concentration of the active ingredient in two batches of pyriproxyfen from different manufacturing date and expiration dates in the laboratory; and 4) determine the efficacy of pyriproxyfen over three months in water from different types of domestic water supplies. These objectives were addressed in four separate experiments corresponding to the stated objectives. The first three experiments: residual effectiveness in the field; batch effectiveness; and active ingredients were carried out in the context of a cluster randomized controlled trial aiming to assess a pyriproxyfen intervention in reducing mosquito infestation and dengue transmission [18]. The fourth experiment on water quality was set in Prachuap Khiri Khan province investigating whether differences in water quality and sources could impact on the effectiveness and persistence of pyriproxyfen. Such information is important to guide vector control operations, hence ensuring adequate use of pyriproxyfen in different environments.

## Methods

### Ethics statement

The project was approved by the Khon Kaen University Ethics Committee (KKUEC) (Record No. 4.4.01: 29/2017, Reference No. HE601221, 1 September 2017); the London School of Hygiene and Tropical Medicine Ethical Committee, UK (LSHTM Ethics Ref: 14275, 16 August 2017); the Regional Committee for Medical and Health Research Ethics, Section B, South East Norway (REK Ethics ref: 2017/1826b, 03 March 2018); the Ethics Committee of the Faculty of Tropical Medicine (MUTM Ethics Ref: 2020-045-1, 21 July 2020); and the Ethics Committee of the Faculty of Tropical Medicine—Animal Care and Use Committee (FTM-ACUC Ethics Ref: 023/2020, 22 July 2020).

### Study site

This study was conducted in two areas

1) Northeast Thailand: field work was carried out from September 2019 to January 2020 in Khon Kaen district (S1 Fig) in households included in the treatment arm of the cluster randomized controlled trial (RCT), but after the trial was completed. Details of the trial, on which the current study is based, have been described elsewhere [18]. Briefly, the trial was carried out in Khon Kaen and Roi Et urban districts in northeastern Thailand during 2018–2019, where 18 out of 36 clusters, 10 houses per cluster, were treated with pyriproxyfen, with nine treatment clusters in each district. Pyriproxyfen was applied to all permanent indoor and outdoor household containers that contained water. The recommended WHO dose of 0.01 mg/L active ingredient (a.i.) pyriproxyfen was used with a single treatment applied every 3 months [20] according to manufacturer’s guidelines starting in June 2018, followed by treatments in September 2018, December 2018, March 2019, and June 2019. For these treatments Sumilarv 0.5G sachets (Sumitomo Chemical Group Co. Japan) were used with an active ingredient content of 0.5% w/w, manufactured in July 2016 and expired in July 2019 (batch no. 6710F4).
2) In Western Thailand, the effectiveness of pyriproxyfen in various water sources was assessed outside of the RCT. Prachuap Khiri Khan is situated along the western coastline of the Gulf of Thailand (S1 Fig), with a total area of 6,368 km^2^ encompassing agricultural fields (45%), forested areas (42%), and residential areas (7%). Due to rural development initiatives, basic infrastructure such as main roads, electricity, and tap water are now prevalent throughout the province, fostering tourism and industrialization, particularly in production of preserved fruits. Economic growth has reshaped social dynamics, transforming rural areas into semi-urban and urban areas. These social changes, coupled with a decentralized dengue control policy, may have contributed to increased dengue incidence. From 2005 to 2009, the Ministry of Public Health, Thailand, designated Prachuap Khiri Khan as a high-risk area for dengue [21]. The province features an earth dam that blocks the Pran Buri River, forming a large reservoir. Processed water from the reservoir is distributed as tap water by the Provincial Waterworks Authority (PWA) to urban and some semi-urban areas. However, water management in rural and semi-urban areas falls under the purview of subdistrict administrative organizations. Nong Ta Team subdistrict, Pran Buri District, Prachuap Khiri Khan was chosen for water collection due to its semi-urban nature and diverse water supply sources.

### Residual effectiveness in the field

**Fig 1:**
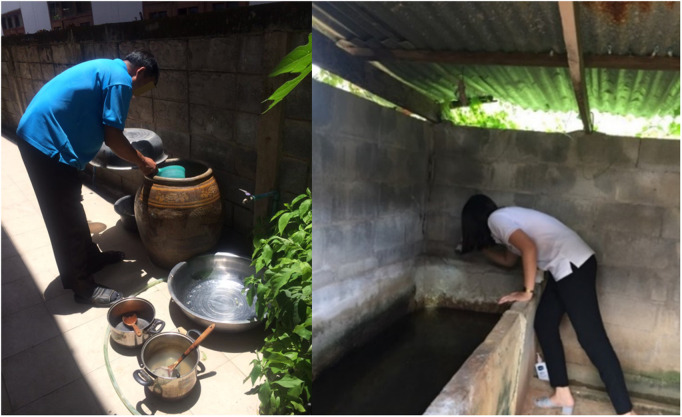
Left panel: Example of medium-sized round dragon jar (ca. < 50 cm height). Right panel: Example of large-sized square containers (ca. > 50 cm in height and ca. > 100 cm long). Photo credits by authors.

Key container types. A key container type is defined here as a category of container that is generally mosquito positive, producing high numbers of immatures (larvae and pupae) per container. Key container types were determined using entomological data collected during 2017–2019 from a total of 180 households in Khon Kaen. The key container determination was based on proportion of container types that were mosquito positive and mean number of immatures per container. Two types of key containers were identified: a) medium-sized round-shaped, glazed, fired, earthen ‘dragon’ jars and b) large-sized square-shaped cement containers (Fig 1). Ten houses were randomly selected from five of the nine clusters included in the intervention arm of the RCT. One container of each type was selected in each household, resulting in 20 key containers for treatment and follow-up (Table 1). Container selection was done by convenience sampling in houses that had both dragon jars and tanks and whose household owners allowed water to be collected during the study period.

**Table 1:** General container characteristics of 20 containers used in the study.

	Round ‘dragon’ jars (n = 10)	Square water holding tanks (n = 10)
**Size**	Medium; < 50 cm in height	Large; > 50 cm height; > 100 cm length
**Use**	Gardening; washing clothes, dishes, hands, and vegetables for food	Mainly for bathing (one tank used for washing clothes)
**Material**	Baked (fired/glazed) clay	Cement
**Color**	Brown	Gray
**Frequency of use**	6 jars used daily; 4 used at least once weekly	All used daily
**Location**	Outdoors (3 under roofing)	All in bathroom
**Exposure to sun**	Partially shaded/ sunlit	Shaded (indoors)
**Cover (lid)**	All, except one uncovered	All uncovered
**Water source**	Piped water	Piped water
**Water condition**	Clear (one with minimal algal growth)	Clear

### Container treatment with pyriproxyfen

These 20 containers were treated on 29 September 2019 with 0.01 mg/L active ingredient pyriproxyfen following WHO recommendations [7]. The pyriproxyfen used was Sumilarv 0.5G (Sumitomo Chemical Group Co. Japan) with an active ingredient content of 0.52% w/w and a manufacture date of March 2018 and expiry date of March 2020 (batch no.’s 8305F4 and 8306F4). These treatments were done after the cluster randomized controlled trial (RCT) had finished in June 2019.

### Water collections

Water samples were collected at eight time points, once before treatment and seven times after treatment (Table 2), from each of the 20 selected containers using a water dipper and a measuring cylinder. Water was collected between 8.00 and 11.00 am to ensure consistency in sampling conditions. At each time point, 1500 ml of water was taken from each container. For convenience this was divided between 15 samples, each taken after stirring the container. Water samples were stored in clean (sterile) sealable plastic bags (Whirl-Pak. Nasco, Madison, WI, USA) and were sent to the Department of Entomology, Faculty of Tropical Medicine, Mahidol University, Bangkok where larval bioassays were conducted within 4–5 days of sample collection. At each time point, the temperature, pH, conductivity, total dissolved solids (TDS), and salinity of the water were measured in each container.

**Table 2:** Times of water collections in the field after treatment with pyriproxyfen and start of larval bioassays in laboratory. T-1 = before treatment. T0 = within 24 hours after treatment.

	Water collections in the field post-treatment		Start of bioassays	Containers last treated
Time	Days after treatment	Collection date	Date	Month
T-1	–	29-Sep-2019	1-Oct-2019	June 2019
Pyriproxyfen treatment	–	29-Sep-2019	–	
T0	1 (24Hr)	30-Sep-2019	1-Oct-2019	Sep 2019
T1	16	15-Oct-2019	18-Oct-2019	Sep 2019
T2	30	29-Oct-2019	1-Nov-2019	Sep 2019
T3	47	15-Nov-2019	18-Nov-2019	Sep 2019
T4	61	29-Nov-2019	3-Dec-2019	Sep 2019
T5	75	13-Dec-2019	16-Dec-2019	Sep 2019
T6	96	3-Jan-2020	6-Jan-2020	Sep 2019

### Larval bioassays

Larval bioassays were performed on 3^rd^ instar laboratory-reared *Ae. aegypti* larvae (Bora-Bora strain F57, susceptible strain) exposed to the field-collected water, according to WHO guidelines [22]. For each bioassay, four plastic test cups (450 ml) filled with 200 mL (5–10 cm depth) of water from the field and four cups of commercial bottled water serving as untreated (negative) controls. Twenty larvae were placed in each cup using a clean pipette. Larval and pupal mortalities were observed daily until emergence of adults. Any physical deformities that occurred in either the successive development (growth, molting) of instars or in emerging adults were observed. Water from each container was tested. The experiment terminated when all control larvae or pupae had either died or successfully emerged as adults.

At the end of the observation period (approximately 10–15 days), pyriproxyfen impact was expressed as percentage of inhibition of emergence (IE) based on the number of larvae that failed to develop and emerge successfully into viable adults by counting both adults and empty pupal cases (see data analysis section below for more details). Moribund and dead larvae and pupae, as well as adult mosquitoes not completely separated from the pupal case were recorded as ‘not having survived’. The time duration of the test required larvae be provided with a small amount of food (ground fish food in suspension) at a concentration of 10 mg/l at two-day intervals until final counts were made. The powdered food was suspended in water to make a slurry and one or two drops added per cup. Each cup was covered with nylon netting to prevent escape of emerging adults. The test containers were held at 25–28°C with a 12 h photoperiod light:dark cycle.

### Batch effectiveness

The applied pyriproxyfen came in two different packaging formats: granules in teabag sachets and granules in bulk packaged 1-kg bags. The teabag sachets were used in the RCT and bulk granules were used in residual effectiveness in the field (during T-1 to T0). We evaluated the IE of both packaging types using the same bioassay procedures as described above and using the reverse osmosis water in this experiment. The tea bag sachets were manufactured in July 2016 with an expiry date of July 2019 (batch 6710F4). Two batches of granules both manufactured in March 2018 had an expiry date of March 2020 (Table 3). Bioassays were repeated three times (100 larvae per time) for each batch in a similar manner as described above. Bioassays were performed at the Department of Entomology, Faculty of Tropical Medicine, Mahidol University.

**Table 3:** Compounds and formulations analyzed.

Compound/ Formulation	Sumilarv 0.5 Sachet	Sumilarv 0.5G
**Manufacturer**	Sumitomo Chemical	Sumitomo Chemical
**Formulation**	Sachets	Granules
**Batch no.**	6710F4	8305F4 and 8306F4
**Applied in**	Trial	Residual effectiveness in the field experiment
**Active ingredient according to manufacturer**	0.5% w/w	0.52% w/w
**Manufacturing date**	July 2016	March 2018
**Expiry date**	July 2019	March 2020
**Emergence inhibition test dates**	1 Oct./18 Oct./ 4 Nov. 2019	1 Oct./18 Oct./ 4 Nov. 2019

### Active ingredients

In this experiment the concentration of active ingredient in two formulations (three batches) of pyriproxyfen with different manufacture and expiry dates were assessed (Table 3). Analyses were performed by two mutually independent laboratories. Tests were carried out in July 2020 by Sumitomo Chemical Environ-Agro Asia Pacific SDN BHD, Singapore and in November 2020 by Central Laboratory (Thailand) Co. Ltd., Chachoengsao branch, Thailand. Two replicates of each batch were processed. Both companies used the CIPAC method 715/GR/M with minor in-house modifications to analyze the pyriproxyfen compounds [23].

### Water source and quality

The manufacturer recommends using 0.01 mg/L active ingredient (a.i.) pyriproxyfen (applied as a 0.5% granule formulation). However, water qualities like turbidity and pollution are criteria for dose adjustment [24].

Water was collected from ‘dragon’ jars (Fig 1, left panel) containing water from four different sources: 1) Government tap water 2) Local tap water, 3) Rainwater, and 4) Groundwater. The jars were selected randomly from households in the study site. Government tap water provision and quality is overseen by the Provincial Waterworks Authority (PWA). Water is pumped from a dam nearby, followed by adding alum and quicklime. Sedimentation tanks allow particles to settle, while filters remove impurities. Chlorine is then added to transform the water into a clean, safe resource for communities [25]. Local tap water is provided by the subdistrict administrative organizations and quality treatment is typically simplified. Rainwater is generally harvested locally from roofs and other water contraptions and stored in dragon jars (Fig 1). Groundwater in the study area is extracted using pumping systems. Wells are equipped with pumps, which vary in type depending on the well’s depth and the required water volume. For daily use, people typically pump groundwater and store it in jars.

Water was collected using 1L NASCO Whirlpak plastic bags and water quality recorded including temperature, conductivity, pH, TDS and salinity. Water was brought back to the Department of Medical Entomology, Faculty of Tropical Medicine, Mahidol University and treated with pyriproxyfen using doses recommended by WHO for *Aedes* immature mosquito control as described above. Bioassays were repeated three times using 100 larvae per time in a similar manner as described in Residual effectiveness in the field. New sets of larvae were changed every two weeks and repeated until 13 weeks.

### Data analysis

#### Inhibition of adult emergence (IE).

The percent inhibition emergence (IE%) was calculated for residual effectiveness in the field, batch effectiveness and water quality using the following formula [27]:


$$IE\%=\text{1}\text{0}\text{0}-\left(\frac{T\;\times \text{1}\text{0}\text{0}}{C}\right),$$

where *T* = percentage survival or emergence in treated batches and *C* = percentage survival or emergence in the control. Adult emergence in the controls was never less than 80%, so no tests were discarded. Similarly, controls generally produced 100% emergence (out of all 160 tests, only 9 gave 99% emergence and 1 test gave 97% emergence) so no correction such as Abbot’s [26] was required.

#### Difference in emergence inhibition between container types and time points.

In residual effectiveness in the field we used summary measures for the statistical analysis [27], rather than repeated measures analysis of variance (ANOVA) [28]. Most data values were close to zero at later times, inconsistent with the sphericity (constant variance) assumption of the ANOVA [28], meaning that its use would not have been reliable. So, in terms of summary measures, we summarized the IE as the area under its curve (AUC) over time, from day 1 onwards. Lower values of AUC indicate lower IE over time. This AUC was then compared by the non-parametric Mann-Whitney test between container types (jar or tank), and between daily and weekly frequency of use.

Variables such as temperature, which change over time, were compared with deviations from the expected value of IE over time for that container type, to account for the pronounced general decrease in IE over time. This decrease was modelled as a logistic curve [29]. The expected value was obtained from one fitted logistic curve for each container type, with the upper asymptote (value approached as time tends to positive infinity) being set to zero, and the following three parameters being fitted: lower asymptote (value approached as time tends to negative infinity), the steepness of the slope, and the time corresponding to IE halfway between the upper and lower asymptotes [29] (S2 Fig). The time to reach 50% IE was estimated from this curve, and its confidence interval estimated by bootstrapping [30] over containers with 1000 replicates. Then, for temperature and the measures of water quality, and for each container, the Spearman correlation [31] was calculated with the deviations from the expected value from the logistic curve. Finally, the global association was estimated by combining the observed container-level correlations and standard errors [32] by meta-analysis methods [33], i.e., the container-level results were combined in the same way as study-level results in a meta-analysis.

## Results

### Residual effectiveness in the field

**Fig 2:**
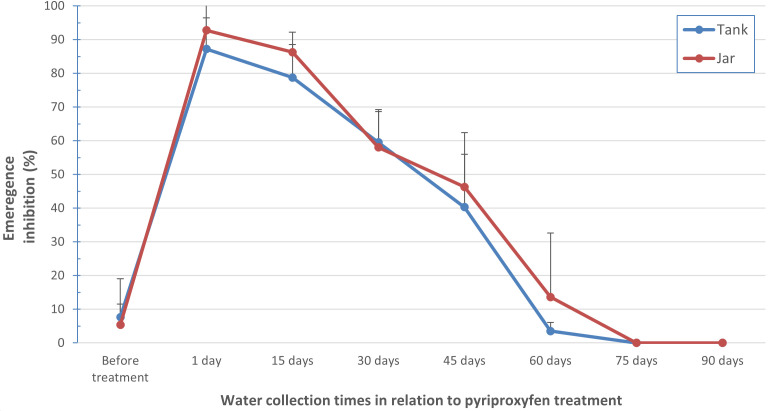
Emergence inhibition of *Aedes aegypti* larvae exposed to pyriproxyfen in water collected from jars and tanks at different time points in Khon Kaen province. Error bars show 95% confidence intervals. The first time point was not included in the analyses which modelled the changes over time as logistic curves.

Overall, the IE in water collected from two types of containers increased from less than 10% before treatment to approximately 90% the same day as the containers were treated (Fig 2).

Overall, the results from the two container types were similar, showing a rapid reduction of IE with less 50% inhibition after 45 days and less than 15% inhibition after two months. Based on the logistic curves, time to reach an IE of 50% was 38.1 days (95% CI 31.5-48.4) for tanks and 39.4 days (95% CI 33.3-50.0) for jars. The corresponding times for 10% IE were 62.5 days (95% CI 55.9-65.4) for tanks and 68.9 days (95% CI 58.6-77.6) for jars.

The areas under the curve (AUC) were similar between the two container types. For the 10 tanks, the median was 3344 percent-days (range 2558–4825) and for the 10 jars it was 3665 (range 2632–5712), *p* = 0.48 by Mann-Whitney test. In terms of frequency of use, the four containers with weekly use had lower AUC (median 2988, range 2632–3519) than the 16 containers that were used daily (median 3651, range 2558–5712), although this was not statistically significant (*p* = 0.08, Mann-Whitney test).

Over the experimental time period, the water quality parameters across both tanks and jars were: mean water temperature was 27.6°C (range 25.0-33.7°C), pH was 7.24 (6.59-8.66), conductivity 708 µS/cm (164–2008 µS/cm), TDS 370 mg/L (197–1010 mg/L), and salinity 0.28 g/L (0.11-0.67 g/L) (Table 4).

**Table 4:** Water characteristics of jar and tank ±standard deviation (SD) and range (minimum- maximum) from difference sources of water, including temperature (°C), pH, total dissolved solids (TDS, mg/L) conductivity (µS/cm), and salinity (g/L).

	Temperature	pH	Conductivity	TDS	Salinity
Tank	27.47 (25.00-33.50 ± 1.76)	7.23 (6.59-7.86 ± 0.28)	745.75 (468-2008 ± 314.44)	387.13 (231-1010 ± 183.33)	0.29 (0.18-0.58 ± 0.10)
Jar	27.7 (25.20-33.70 ± 1.91)	7.25 (6.63-8.66 ± 0.32)	671.31 (164-1582 ± 222.71)	351.99 (197-787 ± 126.65)	0.27 (0.11-0.67 ± 0.11)

**Fig 3:**
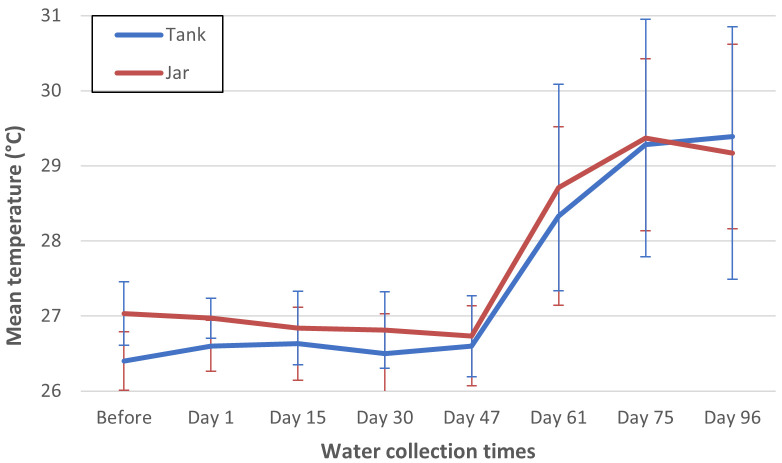
Water temperatures recorded in treated jars and tanks during the study period in Khon Kaen province. Values are presented as mean (with 95% confidence interval error bars), with measurements taken at regular intervals to assess environmental conditions influencing pyriproxyfen efficacy.

Water temperature in the containers increased markedly at day 47 after pyriproxyfen treatment (Fig 3). There was a positive correlation between water temperature and deviations from the fitted values of IE, with the average correlation being 0.203 (95% CI 0.009-0.396, *p* = 0.04). The other correlations were smaller and not statistically significant: for pH, -0.167 (95% CI -0.395 to 0.061, *p* = 0.15); conductivity 0.022 (95% CI -0.218 to 0.261, *p* = 0.859); total dissolved solids -0.040 (95% CI -0.292 to 0.213, *p* = 0.758); salts -0.001 (95% CI -0.241 to 0.239, *p* = 0.994).

### Batch effectiveness

The bioassay test showed that the sachets with an expiry date in July 2019 inhibited 86.3% of mosquito emergence (85%, 86%, and 88%, for each of the three test dates, respectively). The granules with an expiry date in March 2020 inhibited 100% of mosquito emergence (100% inhibition for each of the three dates).

### Active ingredients

Results from the two laboratories showed that the active ingredient in both sachets and granules complied with the manufacturer’s claims (within the 25% acceptable variation of active ingredient) (Table 5).

**Table 5:** Active ingredients (% w/w) in Sumilarv 0.5 Sachets and Sumilarv 0.5 G determined by two laboratories (range in parentheses).

		Active ingredient		
Product	Manufacture date - expiration date	Declared	Sumitomo Laboratory	Central Laboratory
**Sumilarv 0.5 Sachet**	July 2016 - July 2019	0.50	0.481 (0.483-0.478)	0.511 (0.375-0.625)
**Sumilarv 0.5G**	March 2018 - March 2020	0.52	0.448 (0.447-0.449)	0.524 (0.375-0.625)

### Water source and quality

**Fig 4:**
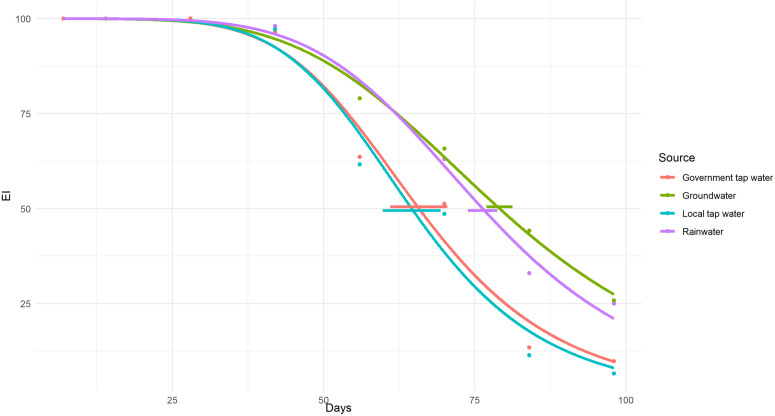
Emergence inhibition (EI) of *Aedes aegypti* larvae exposed to pyriproxyfen in water collected from four different source of water at different time points. Each horizontal line is the 95% confidence interval for the time (number of days) at which 50% EI is reached.

Six weeks (42 days) after treatment with pyriproxyfen, rainwater and groundwater began to show higher emergence inhibition than local and government tap water (Fig 4).

The mean water temperature was 25.24°C (range 24.1-26.5°C), conductivity: 858.41 µS/cm (31–2,480 µS/cm), pH: 7.31 (6.82-7.69), TDS: 426.72 mg/L (14–1,230 mg/L), and salinity: 0.04 g/L (0-0.12 g/L) across all water sources. There were no substantial variations in water temperature between water sources. Both rain and groundwater had the same pH level, while local and government tap water exhibited higher pH levels. Rainwater had the lowest TDS, conductivity, and salinity levels, whereas groundwater had the highest levels for all these three parameters (Table 6).

**Table 6:** Water characteristics ±standard deviation (SD) and range (maximum-minimum) from difference sources of water, including temperature (°C), pH, total dissolved solids (TDS, mg/L) conductivity (µS/cm), and salinity (g/L).

Source of water	Temperature	pH	TDS	Conductivity	Salinity
Government tap water	25.56 ± 0.58 (24.8-26.5)	7.60 ± 0.01 (7.59-7.63)	278.13 ± 1.31 (276-280)	551.53 (548-556 ± 2.77)	0.02 ± 0 (0.02-0.02)
Local tap water	25.21 ± 0.32 (24.8-25.8)	7.66 ± 0.02 (7.63-7.69)	184.63 ± 0.98 (183-187)	375.33 ± 1.01 (374-377)	0.01 ± 0 (0.01-0.01)
Rainwater	24.38 ± 0.18 (24.1-24.8)	6.99 ± 0.07 (6.82-7.10)	15.10 ± 0.65 (14.00-16.00)	32.9 ± 1.30 (31-34)	0.00 ± 0 (0.00-0.00)
Groundwater	25.82 ± 0.2 (25.5-26.1)	6.99 ± 0.03 (6.91-7.03)	1,229 ± 1.34 (1,226-1,230)	2,473.87 ± 2.25 (2,472-2,480)	0.12 ± 0 (0.12-0.12)

There was a significant negative relationship between water pH and IE (F(1,118) = 5.626, p < 0.001). The R^2^ was 0.979, indicating that water pH explained approximately 98% of the variance in IE. Salinity, conductivity, and TDS were positively correlated with IE (F(1,118) = 48.302, p < 0.001; F(1,118) = 37.022, p < 0.001; F(1,118) = 36.699, p < 0.001, respectively). Only temperature was not significant in our model (F(1,118) = 2.301, p = 0.132).

## Discussion

The effectiveness of pyriproxyfen as a larvicide in mosquito control is well-documented, disrupting mosquito development while causing low toxicity to non-target organisms. Its long-lasting residual activity makes it a valuable tool in integrated mosquito management. Understanding the operational effectiveness of pyriproxyfen and the factors influencing its performance in the field are crucial for optimizing its use by national programs. This knowledge helps tailor mosquito control strategies to specific contexts, ensuring responsible use in combating mosquito-borne diseases. Our results contribute to this essential knowledge base.

### Residual effectiveness in the field

The striking decline in pyriproxyfen effectiveness observed in our study suggests there could be potential quality issues of the pyriproxyfen batches used in our experiment, e.g., lowered action or expiration of the active ingredient or limitations in the implementation of the intervention or human behaviors. The lowered action observed in this study, was also found in our RCT as reported in Overgaard et al. 2024 [19]. The frequency of pyriproxyfen treatments is also a critical aspect of its effectiveness in mosquito control. Research and field studies emphasize the importance of adhering to a systematic treatment schedule to disrupt the mosquito life cycle consistently. The recommended treatment frequency for pyriproxyfen applications often depends on factors such as the mosquito species [34,35], the formulation [23–38] of the pyriproxyfen product, and environmental conditions [39]. Treatment frequency is specified in the World Health Organization (WHO) guidelines for mosquito control and by the manufacturer of Sumilarv 0.5G. According to the WHO, the recommended dose of pyriproxyfen is 0.01 mg/L active ingredient and the manufacturer recommends application every 3 months [12,20]. In our study, there was a dramatic decrease in emergence inhibition of mosquitoes over time, with an IE of less than 60% after 30 days and less than 15% after 60 days (far less than the WHO threshold of IE80%), raises important considerations in the context of mosquito control strategies. The emergence of mosquitoes after two months of pyriproxyfen treatment is similar to the one before treatment. The low IE before treatment might have been due to the pyriproxyfen treatment three months earlier in June 2019 as part of the main RCT, although, on the other hand, the experiment showed no more emergence inhibition after 75 days. In any case, these findings contradict earlier research that reported consistent emergence inhibition levels of 90–100% up to 90 days [34,40].

### Batch effectiveness and active ingredient

Mosquitoes exposed to the older batch (July 2019) displayed an average IE of 85%, while those exposed to a newer batch (March 2020) exhibited a higher inhibition rate of 100%. The older batch was tested about 3 months after its expiration, whereas the new batch was tested about 4–5 months before its expiry date (Table 3). Although IE was slightly lower in the older batch than the new one, the active ingredients in both batches aligned well with the specifications of 0.5% w/w. The active ingredients were stable over time and pyriproxyfen appear to remain effective over time, ensuring reliable mosquito control even with older batches. These findings indicate that the pyriproxyfen formulations used in the previous RCT trial [19] and experiments were effective. The observed decrease in mosquito numbers is likely attributed to the residual effectiveness in field containers [41].

Effectiveness of pyriproxyfen could be affected by different extrinsic factors such as water sources, householders’ behaviors and sociocultural practices. In many households, these containers are used for various purposes such as drinking, cooking, and cleaning. People often refill them as needed. In some areas, water may be refilled on a daily basis, and this frequent replenishment of water increases the likelihood of diluting larvicides. In addition, about 48–57% of urban and rural households in northeastern Thailand frequently (weekly) clean water-filled containers in their homes [42], which can also impact on pyriproxyfen residual activity. It is then important to take into account the human behavior in relation to water management and storage practices in assessing the effectiveness of any larvicidal interventions.

### Water source and quality

In this laboratory experiment, the effectiveness of pyriproxyfen in various water sources lasted for 42 days. Specifically, rain and groundwater demonstrated higher emergence inhibition compared to local and government tap water (Fig 4). This discrepancy may be attributed to differences in pH. Rain- and ground water were more acidic than water provided by the local and provincial authorities. We found a negative relationship between water pH and the effectiveness of pyriproxyfen. In mosquito control applications, where pyriproxyfen is used as a larvicide, the pH of the breeding habitat plays a crucial role. Studies have indicated that as the pH level decreases (increasing acidity) [43], the efficacy of pyriproxyfen in inhibiting mosquito emergence tends to decrease. This relationship between pH and pyriproxyfen effectiveness is significant as it underscores the importance of considering environmental factors when implementing larvicidal interventions. Factors such as pH can influence the stability and activity of larvicides. Therefore, in mosquito control programs employing pyriproxyfen, monitoring and adjusting for pH levels of water in breeding sites may be crucial to optimizing the larvicide’s performance.

Additionally, TDS, conductivity and salinity levels of the water sources differed markedly, with rainwater exhibiting the lowest levels and groundwater the highest. These parameters are crucial as they can influence the stability and bioavailability of pyriproxyfen. According to this study high TDS, conductivity and salinity levels in groundwater might enhance the larvicide’s efficacy by facilitating better distribution and contact with mosquito larvae. Conversely, lower TDS, conductivity and salinity levels in rainwater could reduce pyriproxyfen’s degradation, thereby maintaining its potency for a longer duration. We found that TDS demonstrated a positive relationship with IE. However, the association is not straightforward. This ambiguity arises from instances where rainwater, with the lowest TDS, exhibited higher IE compared to tap water, which had higher TDS. The complex relationship between TDS and IE suggests that factors beyond TDS levels alone may influence the effectiveness of larvicides. Further investigation is needed to unravel the complex interactions between TDS and larvicide efficacy in different water sources.

Variations in water quality parameters and their impact on pyriproxyfen’s performance highlight the complexity of implementing mosquito control measures across diverse ecological settings. It is evident that local water characteristics play a critical role in determining the success of pyriproxyfen and any other larvicide for mosquito control. Therefore, tailored strategies that consider these local factors are essential for optimizing the effectiveness of mosquito control programs. The observed decrease in emergence inhibition underscores the necessity for a comprehensive understanding of the dynamics of larvicide application, emphasizing the importance of regular monitoring and timely reapplication. It prompts further research into optimizing application strategies, including dosage adjustments, alternative formulations, or complementary control measures to mitigate the declining effectiveness over time. Moreover, the variation in results highlights the need for region-specific and context-dependent approaches in mosquito control. Factors such as mosquito species composition, local ecology, and human behaviors can influence the effectiveness of larvicidal interventions. Integrating community-based monitoring and adaptive management strategies may be crucial to address the evolving challenges associated with sustained mosquito control.

### Limitations

The study has limitations in sample size and representativeness, particularly in the selection of households participating in the pyriproxyfen-based mosquito control trial. The findings may not fully capture the diversity of socio-economic factors and dengue risk factors present in the study areas. The duration of the study may not have been sufficient to capture long-term trends or fluctuations in mosquito populations and pyriproxyfen effectiveness. Additionally, the study was limited to a single-year dataset, which may not account for seasonal variations in pyriproxyfen efficacy. Multi-year studies would be beneficial to assess the long-term consistency and environmental influences on treatment outcomes. Long-term monitoring could provide more robust insights into the sustained efficacy of pyriproxyfen over time. While the study considered various environmental factors such as water temperature, pH, salinity, conductivity, and TDS, other relevant environmental variables may have been overlooked. Factors like rainfall patterns, vegetation cover were not fully addressed in the study. Addressing these limitations in future research could improve the validity and applicability of findings related to pyriproxyfen effectiveness in mosquito control programs. The current study did not evaluate susceptibility of *Aedes* mosquitoes to pyriproxyfen.

### Future research

The specific factors influencing pyriproxyfen longevity in different containers could provide valuable insights for optimizing mosquito control strategies. It indicates the importance of understanding the intrinsic and extrinsic factors influencing the longevity of the treatment’s impact on mosquito populations. Several factors could contribute to the diminishing effectiveness. Environmental conditions, such as temperature and exposure to sunlight, might influence the stability and persistence of the larvicide [44–46]. Further research is imperative to deepen our understanding of how specific water quality parameters interact with pyriproxyfen’s larvicidal properties. Such studies could inform the development of guidelines for the application of pyriproxyfen in various water conditions, ensuring maximum efficacy and sustainability in mosquito control efforts. A deeper understanding of these factors could aid in the development of improved formulations or application methods to enhance pyriproxyfen’s field performance. Additionally, these insights could support evidence-based decision-making in vector control programs, ensuring more effective and targeted mosquito management strategies. By integrating these findings into practice, public health initiatives can be more effectively tailored to combat mosquito-borne diseases in diverse environmental contexts.

## Conclusion

Laboratory bioassays showed >85% inhibition of mosquito emergence in the laboratory just after treatment. Our study shows a rapid decline in pyriproxyfen effectiveness in the field just 3–4 weeks after the treatment The concentration of active ingredients in the pyriproxyfen batches used in the field complied with manufacturer’s specifications. The reasons for the striking decline in effectiveness over time in the field may be caused by environmental factors, such as pH, water source, and other water characteristics or social factors, such as homeowners’ behaviors and water storage practices. The study emphasizes the need for tailored approaches considering regional, environmental, and social factors and highlights the importance of ongoing monitoring. Despite valuable insights, limitations in sample size and duration of observation were noted. Further research is needed to address these limitations and enhance our understanding of pyriproxyfen’s role in mosquito control. Given the widespread use of pyriproxyfen in public health interventions, these findings underscore the necessity of evaluating its field performance under diverse environmental conditions to ensure its sustained effectiveness in national vector control programs. Strengthening surveillance and community engagement efforts will be crucial in optimizing pyriproxyfen-based mosquito control strategies.

## Supporting information

S1 Appendix Data set 1. Data for residual effectiveness in the field experiment.
(XLSX)

S2 Appendix Data set 2. Data for batch effectiveness experiment.
(XLSX)

S3 Appendix Data set 3. Data for water source and quality experiment.
(CSV)

S1 Fig Map of study locations. The map shows the study site locations where the field trials were conducted. Khon Kaen province in northeastern Thailand and Prachuap Khiri Khan province in central Thailand. Map created by authors using open source QGIS software and datafiles.
(TIFF)

S2 Fig Logistic curve. Logistic curve used to model emergence inhibition over time. It is a sigmoid curve which starts at a high value or asymptote, then declines, gradually approaching a lower value or asymptote (e.g., zero). It is possible to read off the time at which the fitted value is halfway between the two asymptotes.
(TIF)
