# Supplementary figures and images for: Failure of pyriproxyfen at recommended application frequency and doses to control Aedes mosquitoes in Thailand
Source: PLoS Negl Trop Dis. 2025 Dec 22;19(12):e0013042. doi: 10.1371/journal.pntd.0013042 (PMC12721502; doi:10.1371/journal.pntd.0013042)

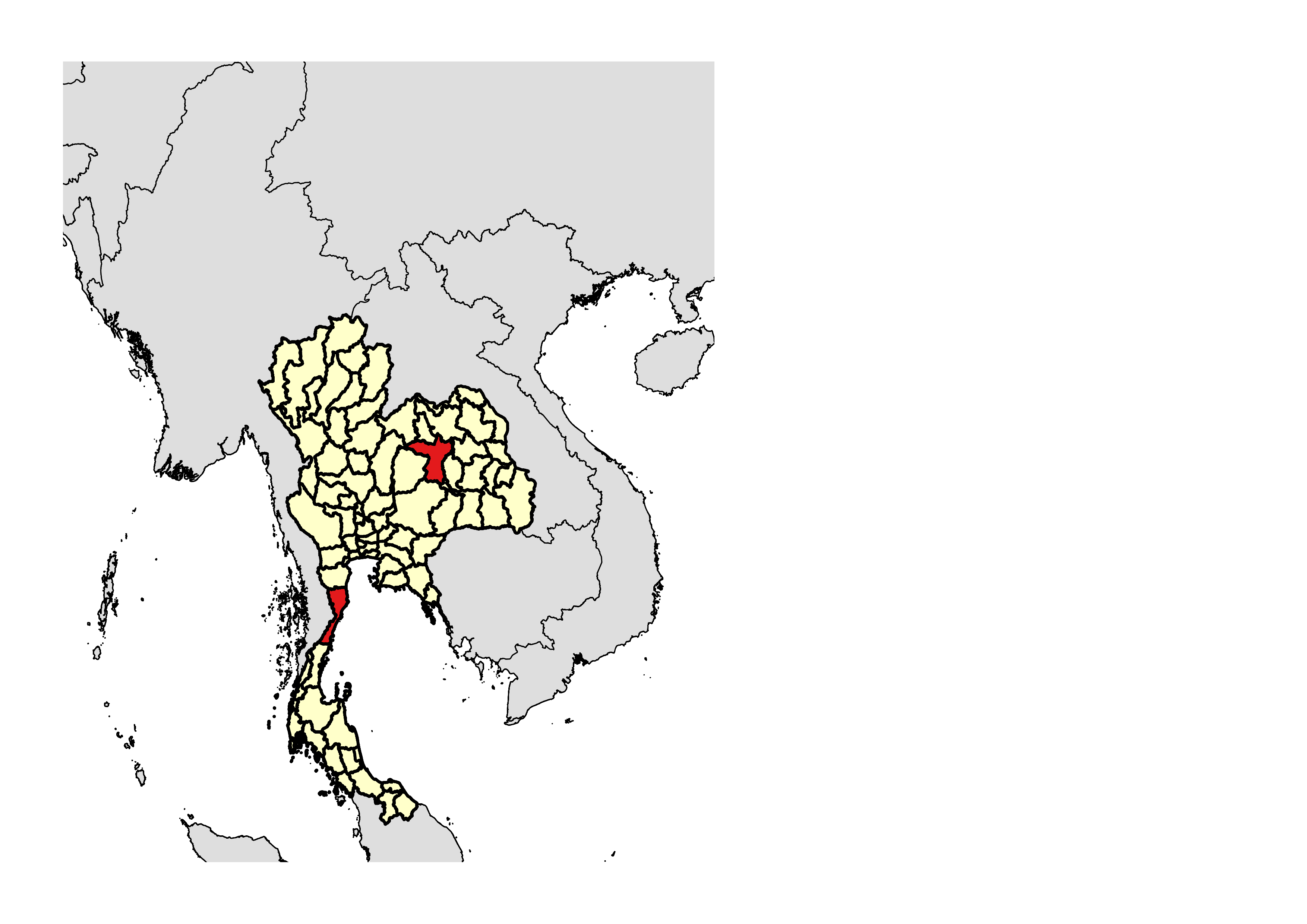

Supplement: S1 Fig — The map shows the study site locations where the field trials were conducted. Khon Kaen province in northeastern Thailand and Prachuap Khiri Khan province in central Thailand. Map created by authors using open source QGIS software and datafiles. (TIFF) [file pntd.0013042.s004.tiff]

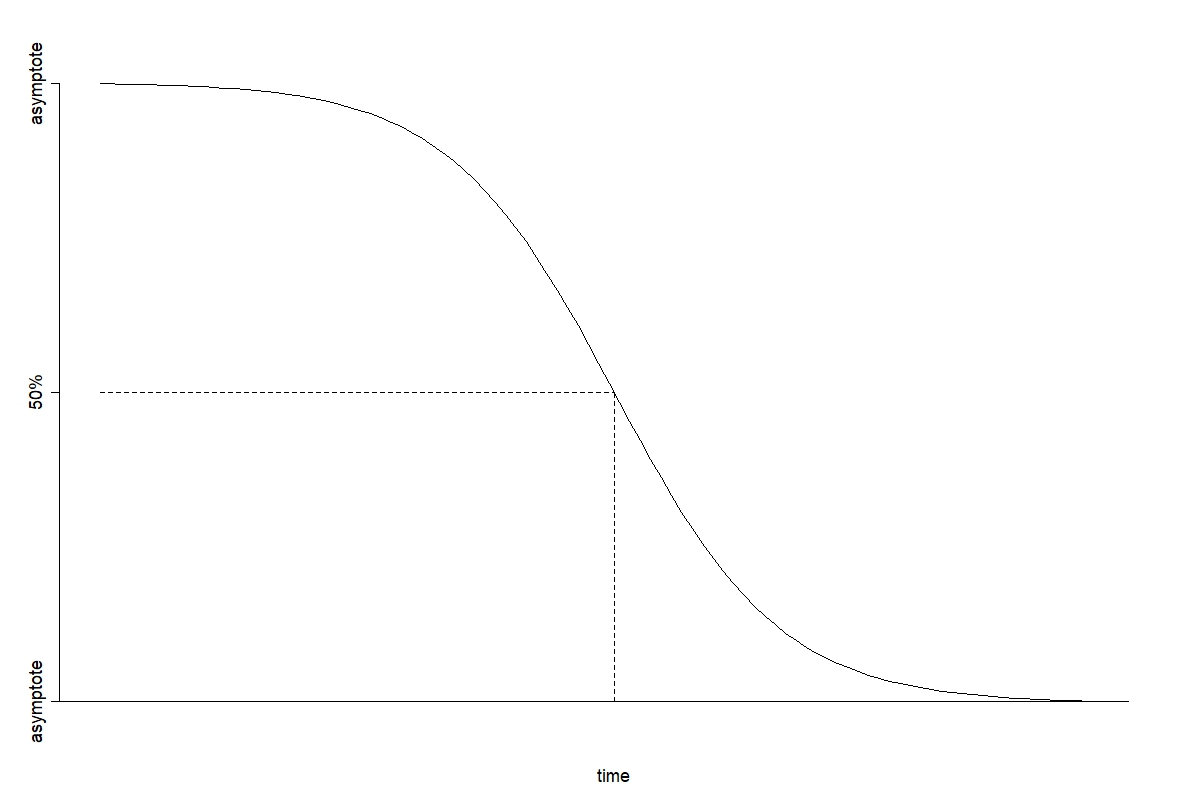

Supplement: S2 Fig — Logistic curve used to model emergence inhibition over time. It is a sigmoid curve which starts at a high value or asymptote, then declines, gradually approaching a lower value or asymptote (e.g., zero). It is possible to read off the time at which the fitted value is halfway between the two asymptotes. (TIF) [file pntd.0013042.s005.tif]
